# Functionally Single-Ventricle Complications After Fontan Palliation—A Narrative Review

**DOI:** 10.3390/jcm15093538

**Published:** 2026-05-06

**Authors:** Małgorzata Kowalczyk, Mirosław Kowalski

**Affiliations:** Department of Adult Congenital Heart Disease, National Institute of Cardiology in Warsaw, 04-628 Warsaw, Poland

**Keywords:** Fontan circulation, functionally single ventricle, long-term complications of Fontan palliation

## Abstract

Functionally single-ventricle (FSV) defects are complex congenital heart anomalies that require Fontan palliation, a surgical procedure redirecting systemic venous blood directly to the pulmonary arteries, bypassing the heart. Despite improvements in surgical techniques and perioperative care leading to enhanced survival rates, patients remain vulnerable to significant long-term complications, due to the unique Fontan circulation physiology. This circulation relies on low pulmonary vascular resistance and preserved single-ventricle function but predisposes patients to venous congestion and reduced cardiac output, resulting in multi-organ dysfunction. Key cardiovascular complications include systolic and diastolic dysfunction of the single ventricle, atrioventricular valve regurgitation, arrhythmias, pulmonary vascular disease, and thromboembolic events. Systemic complications encompass Fontan-associated liver disease (FALD), protein-losing enteropathy (PLE), plastic bronchitis (PB), renal impairment, and endocrine and psychosocial burdens. All the problems induce frequent hospitalizations, psychological challenges, and impaired educational and employment opportunities. Comprehensive management requires multidisciplinary approaches addressing the complex interplay of hemodynamic, organ-specific problems, and psychosocial factors inherent to Fontan physiology.

## 1. Introduction

Functionally single-ventricle defects are a group of congenital heart defects that are not amenable for biventricular repair due to abnormal anatomy of one of the ventricles [1]. Defects classified as single-ventricle include mainly tricuspid atresia (TA), right heart hypoplasia syndrome, including pulmonary valve atresia with an atrioventricular septal defect, Hypoplastic Left Heart Syndrome (HLHS), double-inlet right ventricle, double-inlet left ventricle (DILV), unbalanced atrioventricular canal (AVC) and morphologically undifferentiated, functionally single ventricle [2].

A common feature of various morphologically different FSV-type heart defects is the surgical treatment approach, which involves creating a shunt between the venous flow and the pulmonary arteries, bypassing the functionally single ventricle, regardless of its morphology [3]. Patients disqualified from Fontan palliation or refusing surgery have a poor prognosis [4].

In 1968, Francis Fontan from Bordeaux performed remarkable surgery on patients with tricuspid atresia [5]. He created a connection between the right atrium and pulmonary artery in a patient with a previously performed Glenn connection. The systemic venous return in Fontan circulation goes directly to the pulmonary circulation, bypassing the heart. In the following years, many surgeons improved the Fontan technique. In 1987, Deleval proposed a modification with an intra-atrial tunnel with a right atrial posterior wall that directed venous flow to the pulmonary artery [6]. In 1990, Mareceletti and colleagues reported the use of an extracardiac tunnel, with inferior vena cava flow directed to the pulmonary artery via an external conduit (TCPC—Total cavopulmonary connection) [7]. Improving surgical techniques and peri-operative care resulted in very good outcomes in short-term morbidity and mortality [8,9]. Long-term outcomes are also improving. A 2015 study found 10-, 20-, and 30-year survival rates are 74%, 61%, and 43%, respectively [10]. However, patients are prone to severe complications, which are due to the palliative character of the surgery and the pathophysiology of created circulation. 

## 2. Methodology

This narrative review synthesizes anatomical, physiological, surgical, and failure-management aspects of the Fontan circulation using a purposive, topic-driven selection of key historical reports, guideline documents and contemporary interventional and pharmacological series. The data derives from long-term outcomes and heart failure phenotype studies of Fontan circulation in pediatric and adult cohorts, guidelines and consensus documents on adult congenital heart disease and practical management principles, and research on targeted treatment and rescue strategies in Fontan patients. Articles were identified from reference lists of key reviews and guidelines, and by targeted searches around predefined themes.

## 3. Pathophysiology of Fontan Circulation

The direct connection between the venous system and the pulmonary arteries means that blood flow from the systemic veins to the pulmonary capillaries functions in a portal-circulation-like manner (Figure 1).

The factors determining the efficiency of Fontan circulation are low pulmonary vascular resistance, preserved systolic and diastolic function of the functionally single ventricle, normal systemic vascular resistance, and unobstructed venous return. The effectiveness of venous return bypassing the heart depends on the pressure gradient between the venous system and the pressure in the pulmonary capillaries [11]. The adverse hemodynamic consequences of Fontan circulation include venous congestion and reduced output of the functionally single ventricle [12]. Their consequences may include failure of multiple organs and systems: liver disease secondary to failing Fontan circulation (FALD—Fontan-associated liver disease), chronic kidney disease (CKD), protein-losing enteropathy (PLE), plastic bronchitis (PB), and others.

### Fontan Failure Phenotypes

Fontan circulatory failure may manifest through several distinct pathways, depending on the underlying etiology (Figure 2). Four clinical phenotypes have been described: failure predominantly related to systolic dysfunction (group 1), to diastolic dysfunction (group 2), failure despite apparently normal hemodynamics (group 3), and failure primarily attributable to lymphatic abnormalities (group 4) [13].

The prognosis in Fontan failure depends on cardiac output, pressure in Fontan connection, and pulmonary artery wedge pressure [14]. An overall assessment of patients with suspected Fontan failure should include cardio-pulmonary and systemic complications. The main Fontan complications are presented in Figure 3.

## 4. Cardiovascular and Pulmonary Complications

### 4.1. Single Ventricle Systolic and Diastolic Dysfunction

Remodeling of the functional single ventricle is a key contributor to systolic dysfunction. At the molecular level, univentricular heart development is associated with altered expression of genes involved in myocardial contractility, including downregulation of genes encoding contractile proteins and calcium-handling/adrenergic signaling components (e.g., α-myosin heavy chain, sarcoplasmic reticulum Ca^2+^-ATPase [SERCA], and β-adrenergic receptors) [15,16].

Additional FSV cavity remodeling results from chronic volume overload. Following a systemic-to-pulmonary shunt, FSV preload may increase to approximately 250–350% of the expected value for a left ventricle [17]. This increased preload promotes eccentric hypertrophy, progressive dilation, and spherical remodeling. In contrast, the bidirectional Glenn procedure and subsequent Fontan completion reduce preload (reported as ~80% of normal value for body size), thereby favoring concentric hypertrophy [18]. A further reduction in preload secondary to elevated pulmonary vascular resistance is associated with decreased ventricular compliance and increased filling pressures. Moreover, myocardial architectural remodeling and geometric distortion of the FSV contribute to impaired ventricular contraction [19].

Ventricular interdependence represents an additional determinant of systolic performance, as ventricular contractility depends on interactions between the right and left ventricles. Ventricular hypoplasia may reduce the efficiency of the dominant ventricle [20]. Myocardial perfusion abnormalities also appear to contribute to functional deterioration [21]. Before the Fontan procedure, reduced arterial oxygen saturation may limit oxygen delivery to the subendocardium of the single ventricle [22]. After creating a total cavopulmonary connection (TCPC), a reduction in aortic diastolic pressure—and, consequently, coronary perfusion—has been observed [23]. Studies in complex congenital heart disease populations indicate that right ventricular hypertrophy secondary to systemic pressure loading can impair myocardial perfusion [24,25,26]. Ischemia may occur in the hypertrophied ventricle because of increased metabolic demand (e.g., during exercise), analogous to perfusion–demand mismatch in hypertrophic cardiomyopathy or severe aortic stenosis. Coronary fistulas, which may reduce effective myocardial oxygen delivery, are reported more frequently in patients with an FSV [27]. Collectively, these factors—present in adult patients after Fontan completion and accumulating over time—may promote single-ventricle systolic dysfunction and thereby contribute substantially to heart failure in the Fontan circulation (Figure 4).

The Fontan procedure is also predisposed to diastolic dysfunction through concentric myocardial remodeling [28]. Age-related progression of ventricular structural changes, increased wall stiffness, and a higher proportion of connective tissue are the main contributors to diastolic dysfunction of the FSV. Similar mechanisms (including suboptimal myocyte stretch) have been implicated in heart failure with preserved ejection fraction in older individuals with anatomically normal hearts. The concept of “accelerated aging” of the FSV has been supported by findings reported by Oner et al. [29]. In their study, an FSV ejection fraction <50% was observed in 8% of children during 10 years of follow-up after the Fontan procedure.

### 4.2. Atrioventricular Valve Dysfunction

Atrioventricular valve regurgitation (AVVR) is a common complication in patients with Fontan circulation, leading to increased pulmonary wedge pressure and systemic venous congestion (Figure 5). Large regurgitant volume decreases cardiac output, activating the sympathetic nervous system and augmenting vascular resistance. In long-term observation, AVVR increases the risk of heart failure and arrhythmia, as well as systemic organ dysfunction, and early mortality [30]. AVVR can be caused by structural and functional abnormalities in the valve apparatus and ventricular dysfunction. Patients with FSV of right-ventricular morphology and those with an atrioventricular septal defect (AVSD) have the highest risk of developing AVVR and gradual decline [31]. However, regardless of valve morphology (mitral vs. tricuspid vs. common valve), AVVR is progressive, and linked to poor outcomes. Precise diagnostics should always include various imaging techniques. Transthoracic 2D and 3D echocardiography remains the first-line modality, but followed by transesophageal echocardiography (TEE), cardiac magnetic resonance (CMR), and cardiac catheterization, if needed. Surgical treatment of AVVR is rarely performed, due to high risk and uncertain prognosis after the operation. Surgical valve repair is preferred to valve replacement, unless there is severe valve dysplasia. Unfortunately, surgical treatment does not guarantee a favorable prognosis. Patients with single-ventricle right morphology and a history of tricuspid operation have an 8-year cumulative incidence of death or transplantation of 40% [32]. Patients with ventricular dysfunction, protein-losing enteropathy, and other secondary complications, are at the highest risk of poor outcomes [33].

### 4.3. Arrhythmia

Atrial arrhythmia is a common complication after a Fontan procedure, with prevalence varying by study and surgical technique, in approximately 15% to 60% of patients [32]. The incidence is higher in patients with atriopulmonary Fontan connection compared to lateral or extracardiac tunnel [33]. The core causes of arrhythmia incidence are chronically elevated intra-atrial pressure and intracardiac scars after surgical treatment [34]. Most common atrial tachyarrhythmias in patients with Fontan palliation are intra-atrial re-entrant tachycardia (IART), focal atrial tachycardia, and atrial fibrillation (AF) (Figure 5). Atrial fibrillation is becoming increasingly common in adult patients with Fontan palliation, due to the higher lifespan of Fontan patients [32].

Ventricular arrhythmias (VAs) in patients with a Fontan circulation are less frequent (approximately 11% of patients), but associated with a higher risk of death or heart transplant [35]. Non-sustained ventricular arrhythmias are typically discovered during rhythm monitoring diagnostics. Risk factors of VA include older age at the time of the Fontan procedure and a history of right ventricular-to-pulmonary artery conduit. Treatment can be challenging, due to complex anatomy. Pharmacological treatment is limited, as it is often ineffective or has serious side effects. Rapid hemodynamic decline during arrhythmia requires urgent cardioversion. Despite complex anatomy, catheter ablation should be considered. In selected patients, the implantation of a defibrillator (ICD) is required. A new onset of arrhythmia or decline of a recurrent one should always prompt overall evaluation of the circulatory hemodynamics (Figure 6).

### 4.4. Pulmonary Vascular Disease

The direct dependence of pulmonary vascular resistance on single-ventricle cardiac output has been confirmed in invasive studies [36]. Abnormalities in the embryonic development of the pulmonary vasculature in patients with single-ventricle physiology are thought to determine abnormal structure and function of the pulmonary arteries after birth [37]. The nature of blood flow is also a factor leading to loss of autoregulatory capacity of the pulmonary vascular bed and abnormal vascular tissue architecture. Pulsatile flow through the pulmonary arteries regulates endothelial function, maintains the balance of vasoactive mediator release, influences smooth-muscle remodeling, and promotes uniform recruitment of pulmonary arterioles [38,39]. After completion of the Fontan connection, the protective effects of pulsatile flow on the pulmonary vasculature are absent, having been replaced by an unfavorable continuous flow pattern [40].

Collectively, these factors contribute to a distinct pattern of adverse pulmonary vascular remodeling in patients with long-standing Fontan circulation [41]. The histopathologic changes include eccentric intimal thickening characterized predominantly by acellular fibrosis with collagen deposition [42]. These alterations are associated with reduced pulmonary blood flow and the consequent limitation of cardiac output. Accordingly, the pulmonary vasculature has become a therapeutic target for pharmacologic vasodilation strategies [43].

### 4.5. Thromboembolic Complications

After Fontan palliation, patients have an increased risk of thromboembolism (TE) [36]. All elements of Virchow’s triad are present in Fontan circulation: abnormal non-laminal blood flow, vascular wall injury, and hypercoagulability [44].

Other factors contributing to this elevated risk include the presence of prosthetic materials and arrhythmias [37]. The most severe clinical manifestations of TE are pulmonary embolism and stroke. Thrombus formation occurs mostly in the “right side” of circulation, presenting as venous thrombosis, intra-atrial or Fontan tunnel thrombi, or pulmonary embolism [45] (Figure 7 and Figure 8).

In patients without a subpulmonary pump, pulmonary embolism obstructs the pulmonary vasculature—a primary regulator of cardiac output. Stroke is less common than pulmonary embolism, though recent studies have proved it is often clinically silent and underdiagnosed [46]. Asymptomatic strokes were diagnosed in 39% patients in cerebral magnetic resonance imaging. Both pulmonary embolism and stroke are associated with substantial mortality and morbidity, underscoring the critical importance of thromboprophylaxis. The serious complications associated with TE have led to the recommendation that anticoagulation therapy should be initiated earlier than in the general population. Thromboprophylaxis is indicated mostly in secondary prevention, in patients with a history of TE [2]. However, there is currently no clinical consensus on primary prevention nor the use of non-vitamin K oral anticoagulants.

## 5. Systemic Complications

### 5.1. Fontan-Associated Liver Disease

Fontan-associated liver disease (FALD) represents a major systemic complication in patients who have undergone the Fontan procedure, with a prevalence exceeding 50% in this population [47]. The pathophysiology of FALD is primarily driven by chronic hepatic venous congestion and persistently elevated central venous pressure, which together induce progressive liver injury characterized by fibrosis and cirrhosis [48]. Over time, these pathological changes significantly elevate the risk of hepatocellular carcinoma, necessitating vigilant long-term surveillance [49]. Clinically, FALD may remain asymptomatic for extended periods, complicating timely diagnosis and management. When symptoms do manifest, they often include severe complications such as ascites, variceal bleeding, and hepatic encephalopathy [50] (Figure 9). Accurate diagnosis requires a multimodal approach combining thorough clinical examination with laboratory tests assessing liver function and hematologic status, advanced imaging techniques to evaluate liver architecture and blood flow, non-invasive fibrosis scoring systems, and, in select cases, liver biopsy to confirm histopathological changes [50]. There is no pharmacological treatment of FALD. The use of agents decreasing pulmonary vascular resistance may ameliorate hemodynamics and delay progression of FALD [51]. In many cases, severe FALD is rather an outcome of inefficient Fontan circulation, and it is a presentation of Fontan failure. Patients with end-stage cirrhosis or portal hypertension due to Fontan failure should be considered for combined heart and liver transplant [52].

### 5.2. Protein-Losing Enteropathy

Protein-losing enteropathy (PLE) is another serious systemic complication seen in 5–12% of patients post-Fontan surgery, and is associated with a substantial increase in morbidity and mortality [53]. PLE results from excessive loss of serum proteins into the intestinal lumen, leading to hypoalbuminemia. PLE is related to clinical sequelae such as edema and immunodeficiency [54]. Several risk factors contribute to the development of PLE, including anatomical variations like non-left ventricular morphology, elevated ventricular end-diastolic pressure, and perioperative factors such as renal failure, prolonged cardiopulmonary bypass duration, and extended hospitalization after Fontan surgery [55]. The complex interplay of these factors highlights the need for comprehensive assessment and tailored management strategies to mitigate the incidence and impact of PLE in this vulnerable cohort. The first step in PLE diagnostics is to search for characteristic symptoms and signs (peripheral edema and prolonged infections) along with serum hypoalbuminemia [56]. High alpha-antitrypsin in stool samples confirms a diagnosis of PLE [57]. Multimodality imaging should be implemented for ruling out reversible causes of PLE. In some cases, cardiac catheterization is required to exclude elevated pressure in Fontan connection or to perform catheter interventions [58]. Etiology treatment is important, due to the limited possibility of pharmacotherapy.

### 5.3. Plastic Bronchitis

Plastic bronchitis (PB) presents with a constellation of non-specific respiratory symptoms such as chronic cough, wheezing, and progressively worsening dyspnea, which can easily be mistaken for more common pulmonary conditions. The severity of symptoms often correlates with the degree of airway obstruction caused by the casts, and, in severe cases, respiratory failure may occur. The lifetime cumulative incidence in survivors of the Fontan surgery is about 4% [59]. The increased venous pressure, dysfunction of the bronchial mucosa, and increased pressure in the lymphatic vessels, sometimes with broncho-lymphatic fistulas, are considered to be the main factors involved in the formation of bronchial casts. Due to the rarity and non-specific presentation of the disease, diagnostic vigilance is necessary for timely diagnosis. Evaluation typically involves imaging techniques such as chest X-rays and computed tomography (CT) scans, which may reveal airway obstruction or atelectasis. Ref. [60] states that bronchoscopy plays a crucial role in both diagnosis and management by allowing direct visualization and removal of bronchial casts [61]. Additionally, lymphatic imaging techniques, including dynamic contrast-enhanced magnetic resonance lymphangiography, can identify abnormal lymphatic flow and broncho-lymphatic fistulas, guiding targeted therapeutic interventions [62]. However, patients with a history of PB are at high risk of early death and heart transplant [63]. The onset of PB should prompt further evaluation for hemodynamic alterations or lymphatic abnormalities.

## 6. Other Concerns

Altered hemodynamics and organ-specific comorbidities predispose patients to reduced exercise tolerance [64]. Exercise unmasks a limited single-ventricle output reserve. The absence of a subpulmonary ventricle is responsible for the inappropriately small increase in cardiac output during exertion. Direct connection of the systemic veins to the pulmonary arteries prevents an increase in pulmonary arterial pressure in response to exercise. Impaired regulation of pulmonary vascular resistance contributes to a reduced flow reserve. In individuals with a normal circulation, exercise decreases pulmonary vascular resistance through pulmonary arteriolar dilation and recruitment of additional vessels. In patients with single-ventricle physiology, regulation of pulmonary vascular resistance via these mechanisms is minimal or entirely absent [65].

Venous return is driven by the pressure gradient between the systemic veins and the pulmonary capillaries. The absence of a subpulmonary pump results in chronic venous congestion. Venous pressures below 18–20 mmHg are generally well tolerated. Low pulmonary capillary wedge pressure is also required to maintain venous blood flow. Factors that increase pulmonary capillary pressure (e.g., atrioventricular valve regurgitation, and systolic and/or diastolic dysfunction of the single ventricle) reduce venous return and lead to failure of the Fontan circulation. During exercise, venous pressure and pulmonary blood flow increase only modestly, via the “muscle pump.” Contraction of the lower-extremity muscles and changes in intrathoracic pressure during respiration provide an additional driving force for venous return during exertion, but they do not compensate for the increased preload demand imposed by exercise [66].

Fontan surgery results in stress on renal function. Fontan circulation is associated with both acute kidney injury (AKI) immediately after the operation and progressive, long-term kidney function impairment resulting in chronic kidney disease (CKD). Pathophysiological changes result from incomplete histologic repair after the initial damage during surgery, nephron loss, compensatory glomerular hypertrophy, and chronic interstitial inflammation and fibrosis [67]. AKI is common directly after Fontan surgery, occurring in 16% of patients within 48 h post-operation. The absence of a fenestration and longer duration of bypass are associated with AKI lasting more than 48 h. Prolonged AKI increases hospital length of stay and the risk of sternal wound infection [68].

The long-term impact of Fontan palliation on kidney function is primarily caused by chronically increased pressure in the renal veins and decreased cardiac output, resulting in low blood volume in the renal arteries. This causes renal impairment, measured by GFR < 90 mL/min/1.72 m^2^, in about 10% of patients years after surgery. Chronic kidney disease is associated with higher mortality long after Fontan surgery [69]. Treatment and prophylaxis of renal failure are especially challenging in this population, as most nephroprotective drugs decrease cardiac output through vasodilation and volume loss.

Altered hemodynamics after Fontan surgery also impact the endocrine system. Thyroid, parathormone, and adrenal hormone levels are often outside normal ranges. Growth hormone deficiency and malnutrition contribute to growth retardation. Children with Fontan circulation have mild deficits in height, with greater deficits in those with protein-losing enteropathy (PLE) [70].

All these problems make Fontan patients more prone to psychiatric and socioeconomic issues. Frequent hospital stays, psychological difficulties, and challenges in education make it hard to find and maintain well-paid employment.

## 7. Future Directions

A high risk of complications raises concerns regarding patients’ quality of life. Most adverse effects following surgical repair result from specific hemodynamic alterations, and cannot be fully resolved. Ongoing scientific and clinical efforts aim to mitigate the impact of venous congestion and reduced cardiac output. Physical exercise remains the most effective strategy for maintaining overall health in patients with cardiovascular disease [71]. This approach is similarly beneficial for Fontan patients. Despite their reduced exercise tolerance, physical activity facilitates the adaptation of Fontan circulation to physiological stress and enhances the function of the muscle pump [72]. Improved venous return, supported by well-conditioned leg muscles, can contribute to increased cardiac output.

Recent studies have focused on optimizing physical exercise protocols for Fontan patients. A recent meta-analysis assessed the impact of exercise on peak oxygen consumption in this population, highlighting exercise performance as a modifiable factor [72]. Cardiac rehabilitation should be routinely implemented, introduced early, and tailored individually, based on clinical status and mobility. Numerous study findings challenge the traditional notion that, particularly pediatric patients, require excessive protection from physical activity. Exercise is also employed as a tool to evaluate Fontan circulation, with novel diagnostic methods being integrated. Invasive venous-pressure measurement during cardiopulmonary exercise testing and exercise-induced pulmonary vascular resistance have been demonstrated to be reliable markers for comprehensive assessment of Fontan circulation [73,74].

Recent years have seen increased awareness and considerable efforts toward the earlier diagnosis of Fontan-associated liver disease (FALD). However, the altered pathophysiology of liver congestion and fibrosis in Fontan patients has rendered classic diagnostic schemes and scoring systems less reliable. Non-invasive predictors of severe fibrosis, such as transient elastography combined with platelet count (as utilized in the FonLiver risk score), have demonstrated better predictive accuracy for severe liver fibrosis compared to conventional models [75]. The diagnosis of hepatocellular carcinoma (HCC) within the specific context of FALD is critically important. A recent review emphasized the significance of early detection of carcinogenesis, as it is a major factor contributing to improved survival outcomes [76,77]. Furthermore, new insights into micromolecular changes in the livers of Fontan patients have emerged. A recent study revealed significant metabolic abnormalities, particularly in amino acid metabolism, as well as disruptions in other metabolic pathways [78].

Future research will undoubtedly focus on improving the diagnostics of high pulmonary vascular resistance (PVR) and developing therapeutic interventions aimed at preventing pulmonary vascular disease in this population. The utility of PVR measurement has proven to be a valuable prognostic factor during the subsequent stages of Fontan palliation [79,80,81] Scientists are actively seeking novel pharmacotherapeutic agents that may prevent Fontan circulation failure. The pulmonary vasculature remains the main therapeutic target. Pulmonary vasodilators have been increasingly utilized worldwide, and new agents are being administered in patients with single-ventricle physiology [82,83].

Modern devices, such as left ventricular assist devices (LVADs) or atrial flow regulators, are widely used in patients with normal cardiac anatomy. An increasing number of studies are being published on the use of these devices in patients with congenital heart defects, including those after the Fontan procedure [84,85,86] The atrial flow regulator has been successfully used to create a fenestration between the pulmonary artery and the left atrium, leading to clinical improvement in patients with failing Fontan circulation [87]. Growing experience is being gained with the broader use of LVADs in Fontan patients, where the device is primarily employed as a bridge to transplantation [88,89].

## 8. Conclusions

Fontan surgery remains the sole technique to extend the lifespan of patients with a single-ventricle heart. Nevertheless, it is a palliative procedure associated with numerous short- and long-term complications. During the initial era of Fontan palliation, only patients meeting the so-called “10 commandments” were eligible for the surgery [90]. Currently, a broader patient population undergoes this procedure. This expanded approach offers hope to the majority of patients but concurrently increases the risk of comorbidities and diminished quality of life. Most long-term complications arise from specific circulatory characteristics, namely chronic systemic venous congestion and reduced cardiac output. Advances in diagnostic methods allow for the early detection of cardiovascular and systemic organ failure. While some complications can be managed pharmacologically or through interventional procedures, optimal hemodynamic conditions can only be achieved via heart transplantation. End-organ damage frequently necessitates simultaneous kidney or liver transplantation. Ongoing advances in diagnostics, pharmacotherapy, and mechanical circulatory support offer promising opportunities to improve long-term outcomes and quality of life in this growing patient population.

## Figures and Tables

**Figure 1:**
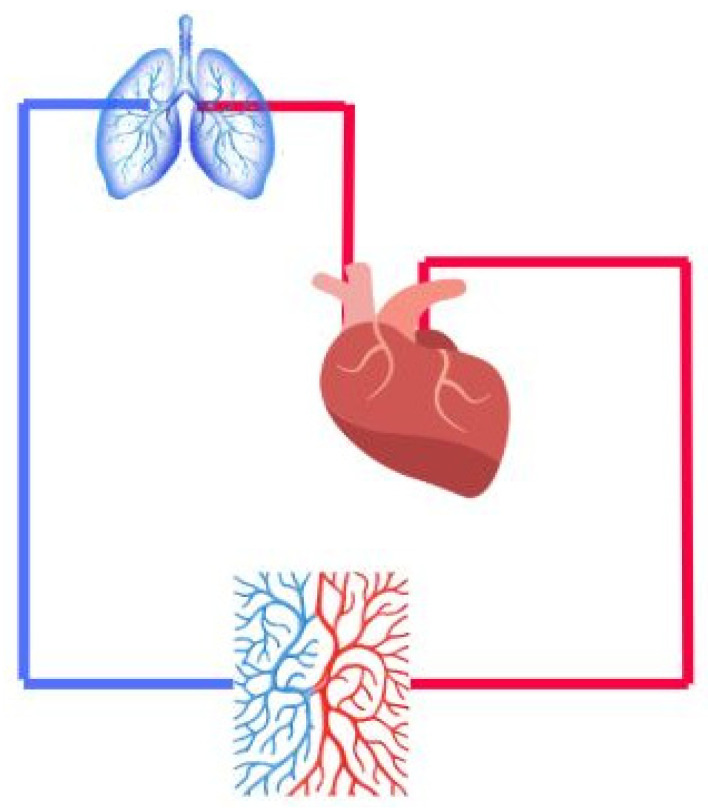
Fontan circulation scheme.

**Figure 2:**
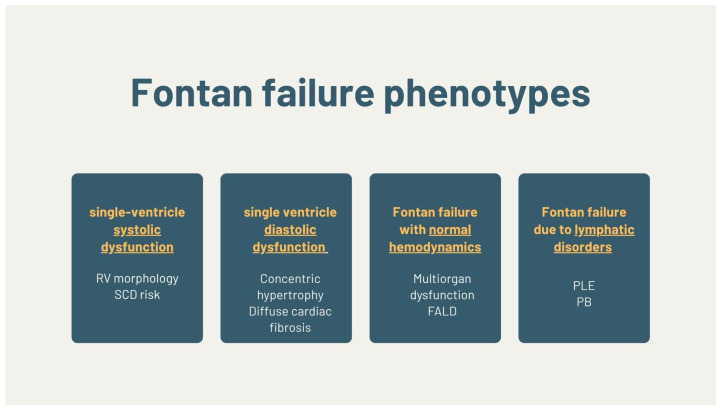
Fontan circulation failure phenotypes. FALD—Fontan-associated liver disease; PB—plastic bronchitis; PLE—protein-losing enteropathy; RV—right ventricle; SCD—sudden cardiac death.

**Figure 3:**
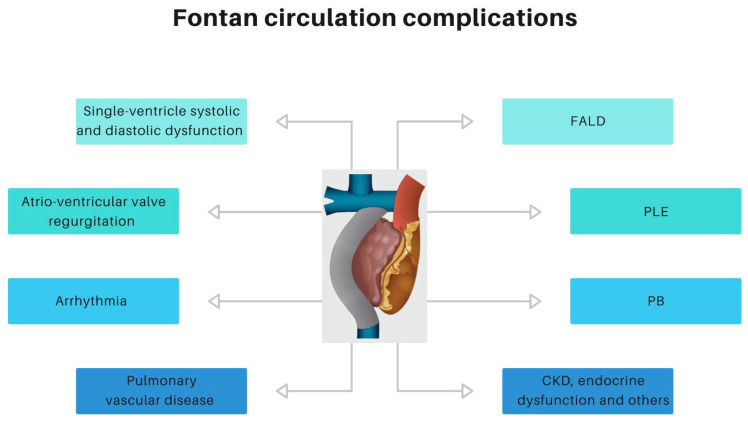
Fontan circulation complications. CKD—chronic kidney disease; FALD—Fontan-associated liver disease; PB—plastic bronchitis; PLE—protein-losing enteropathy.

**Figure 4:**
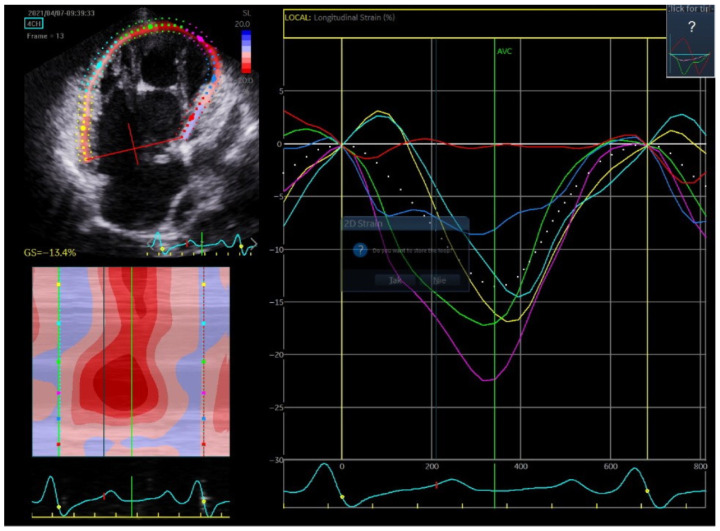
Transthoracic echocardiography, modified four-chamber view, STE technique. Reduced GLS of a dilated, remodeled FSV in a patient with HLHS. HLHS—hypoplastic left heart syndrome; GLS—global longitudinal strain; STE—speckle tracking echocardiography.

**Figure 5:**
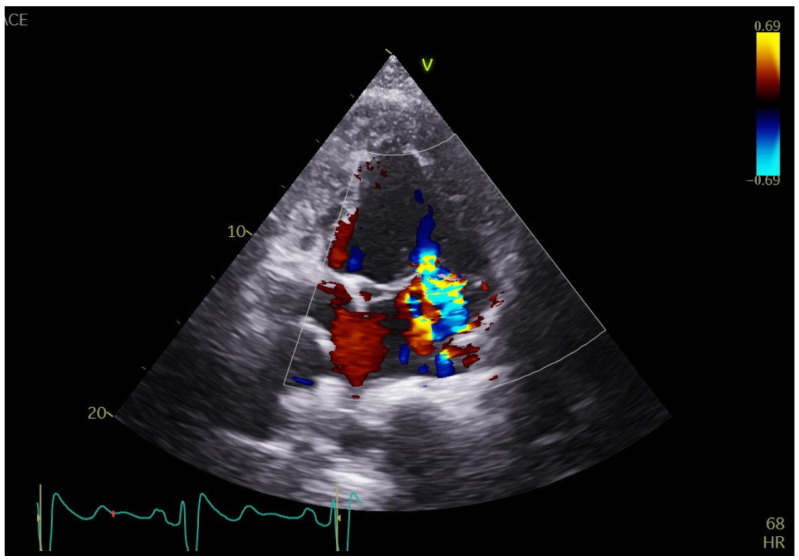
Transthoracic echocardiography, modified 4-chamber view, color Doppler technique. Moderate mitral regurgitation in a patient with tricuspid atresia.

**Figure 6:**
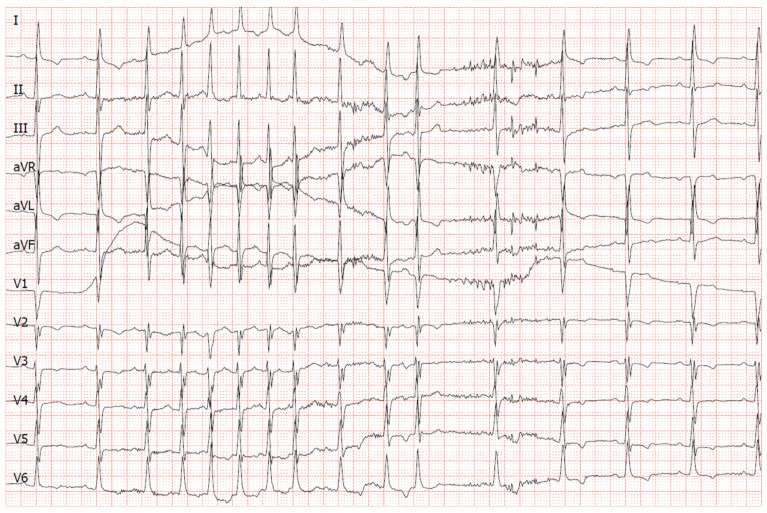
Showing 48 h Holter ECG monitoring. Short atrial tachycardia episode in a patient shortly after ischemic stroke.

**Figure 7:**
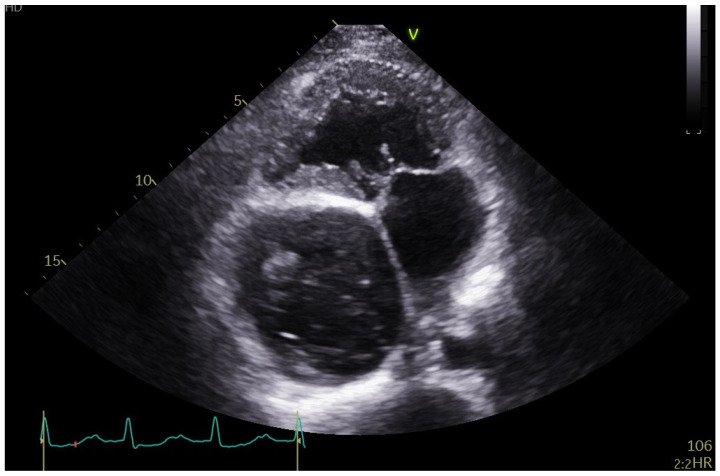
Transthoracic echocardiography modified 4-chamber view, 2D technique. A thrombus and spontaneous echo contrast in the atrium in a patient with atriopulmonary connection.

**Figure 8:**
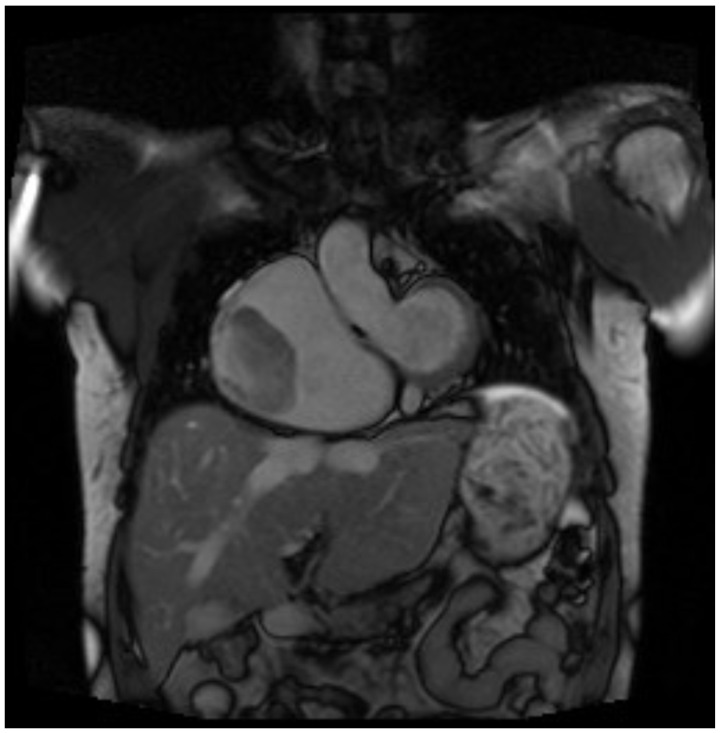
Cardiac magnetic resonance imaging, coronal plane. A large thrombus in the right atrium in a patient with FSV and atrio-pulmonary connection.

**Figure 9:**
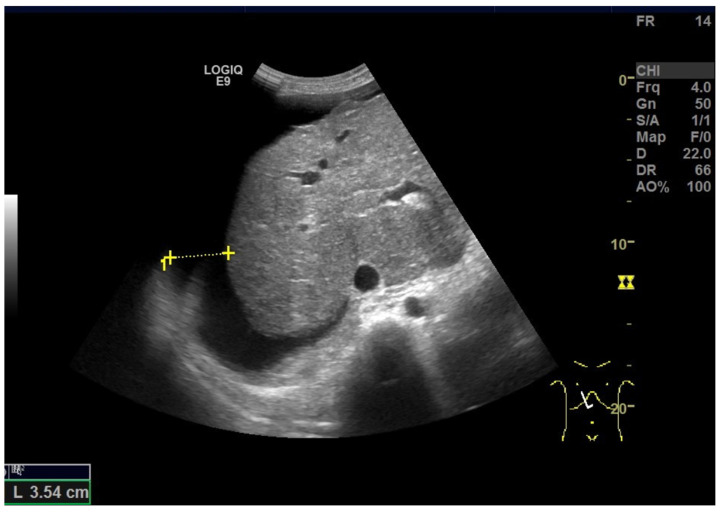
Abdominal ultrasound in a patient with Fontan circulation failure, secondary liver cirrhosis, and massive ascites.

## Data Availability

The data underlying this article will be shared upon request from the corresponding authors.

## Abbreviations

The following abbreviations are used in this manuscript:

AF	Atrial Fibrillation
AVSD	Atrioventricular Septal Defect
AVVR	Atrioventricular Valve Regurgitation
AKI	Acute Kidney Injury
CKD	Chronic Kidney Disease
CT	Computed Tomography
DILV	Double Inlet Left Ventricle
ECG	Electrocardiogram
FALD	Fontan-Associated Liver Disease
FSV	Functionally Single Ventricle
GLS	Global Longitudinal Strain
HCC	Hepatocellular Carcinoma
HLHS	Hypoplastic Left Heart Syndrome
ICD	Implantable Cardioverter Defibrillator
LVADs	Left Ventricular Assist Devices
PB	Plastic Bronchitis
PLE	Protein-Losing Enteropathy
PVR	Pulmonary Vascular Resistance
RV	Right Ventricle
SCD	Sudden Cardiac Death
STE	Speckle Tracking Echocardiography
TA	Tricuspid Atresia
TCPC	Total Cavo Pulmonary Connection
TE	Thromboembolism
TEE	Transesophageal echocardiography
VA	Ventricular Arrhythmia
